# Polarization as a Process: The Potential of Process Ontology for Understanding Cellular Symmetry Breaking

**DOI:** 10.1002/bies.70135

**Published:** 2026-04-09

**Authors:** Marieke M. Glazenburg, Liedewij Laan

**Affiliations:** ^1^ Department of Bionanoscience Delft University of Technology Delft The Netherland

## Abstract

Research in molecular cell biology has typically been focused on identifying specific genes and proteins responsible for cellular phenomena. However, it is increasingly recognized that the function of many biomolecules is variable and context dependent, raising the question if specific components can adequately explain cellular mechanisms. Philosophers of biology have proposed an alternative perspective known as process ontology, posing that not objects or molecules, but processes are the fundamental units of living systems. Process ontology is gaining popularity in biological theory, but remains challenging to integrate into scientific practice. Here, we assess the applicability of the process perspective in the context of a concrete biological system, namely polarization in budding yeast. We identify relevant processes in yeast polarization at different timescales and examine how these processes affect our understanding of polarity. Using this case study, we demonstrate how the processual perspective evokes new kinds of scientific questions and provide concrete pointers for incorporating processual thought into cell biological research.

## Graphical abstract

Process ontology proposes that biological systems are best understood as composed of dynamic processes rather than static components. However, application to concrete research practice has remained challenging. We use cell polarity in budding yeast as a case study to examine the implications of applying process philosophy to cell biological research.

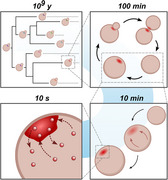

## 1 Introduction

Since the revolution of the modern synthesis [1], cell biology has become strongly focused on understanding cellular functions through molecular mechanisms. Biological phenomena have been extensively studied by identifying related genes and specific protein interactions, often through perturbation experiments, after which a molecular model is proposed with as few necessary elements as possible. While incredibly powerful in the right context, the wealth of biological data available today is revealing an increasingly more complex picture: many cellular functions and phenotypes are not formed by a few dominant, conserved and strongly interacting proteins, but are instead orchestrated by large, heterogeneous and weakly interacting collectives. This complexity raises questions about the extent of applicability of traditional reductionistic approaches and has sparked numerous debates about the role of self‐organization and collectivity in biological systems [2, 3, 4].

In philosophy of science, great progress has been made in the study of alternative perspectives on reality that could be a better fit with biological observations. These discussions stem from the field of ontology, the branch of philosophy that concerns itself with the nature of entities and phenomena [5]. The molecular reductionist point of view relies on an object‐focused ontology, by assuming that the fundamental building blocks of biological systems are primarily static objects in the form of biomolecules, particularly genes and gene products. As a consequence, explanations for biological phenomena are often sought at the level of the specific molecules responsible for a cellular phenotype.

An alternative position that is gaining traction in the philosophy of the life sciences is process ontology. According to process ontology, the world is ultimately made up of dynamics and change; in other words, of processes [6, 7, 8]. A process by definition possesses a temporal dimension and only exists extended in time, rather than objects that are by themselves static. Processes can organize to form stable, object‐like structures, but the stability is limited to specific timescales and often requires work to maintain.

Since many biological phenomena are highly dynamic, a process perspective can be attractive to biologists from a conceptual point of view [9]. However, there is still a gap between philosophers of science advocating processual thought and scientists in the lab: especially molecular cell biology tends to be firmly rooted in object‐based thinking, and application of the abstract principles of process philosophy into actual research practice is not immediately straightforward. To start bridging this gap, we here assess the merits of process thinking in the context of a concrete cell biological case, being cell polarity in the budding yeast *Saccharomyces cerevisiae*, the authors’ primary expertise. What can be gained from applying the principles of process philosophy to budding yeast polarization, and how do these insights translate to cell biology in general?

In the following, we will first provide a brief overview of how process philosophy opposes the traditional object‐focused perspective in general cell biology, and how it matches biological observations. We then focus our attention on budding yeast polarity and describe this system in terms of core processes, separated by timescales, and subsequently discuss the consequences of interpreting polarization as a hierarchy of processes. We finish with a more general perspective on the application of processual thinking in theoretical and experimental research.

## 2 From Objects to Processes in Cell Biology

Current molecular cell biology mostly operates from a perspective centred around specific molecules or objects, to varying degrees of success. From the object‐based point of view, a cell can be considered a large, intricate collection of particles that specifically localize and interact to perform certain functions. To understand how those functions are maintained, the particles themselves typically become the main focus: which gene knockouts produce a certain phenotype, which protein interactions are strongest, which amino acid residues are necessary for enzymatic activity. In other words, explanations are sought in (preferably individual) genes and proteins as the primary components of biological systems. Importantly, the components in this view are (I) isolated from as much context as possible and (II) approximated as temporally stable.

Undeniably, this perspective has its merits. The impressive advances of molecular biology in the past half decade allowed cellular processes to be broken down into simpler pieces and provided extensive knowledge about specific gene products and their interactions, as well as a plethora of molecular tools that are nowadays indispensable. However, for questions concerning higher levels of organization, a strongly object‐focused perspective can become limiting. Recent observations increasingly point in this direction: monogenic traits are the exception rather than the norm [10, 11], protein functions change depending on the chemical environment or the genetic background of the organism [12, 13, 14], and different organismal species can execute a similar cellular function with great genetic variety [15, 16, 17]. Rather than being dominated by only a few relevant components, many biological processes seem to rely much more on collective self‐organization by a wide range of different components [18, 19].

Over the past decades, it has been increasingly recognized that individual genes and proteins are not always the appropriate level of explanation for biological phenomena [20, 21]. Biophysical and soft matter approaches in particular have made great advances in describing cellular processes using non‐equilibrium thermodynamics and collective variables derived from statistical physics [22, 23], such as compartmentalization by liquid‐liquid phase separation in cells [24] or the dynamic instability framework for the description of microtubule dynamics [25]. While successful in their respective areas, translation of these approaches to more complex or heterogeneous cellular phenomena remains challenging. For yeast polarity and other symmetry breaking systems, the study of self‐organization and spontaneous pattern formation has fueled a large body of work based on reaction‐diffusion systems and Turing patterns [26]. Although these efforts have made crucial contributions to the understanding of emergent order from molecular interactions, Turing‐type models are generally based on sets of specific proteins with a fixed number of defined interactions. Hence, typical cell biological research still tends to gravitate towards identification and characterization of specific molecules as the ultimate explanatory units.

Process ontology provides a possible alternative perspective on the interpretation of biological systems. The core claim of process ontology is that processes—dynamic structures characterized by continuous change—rather than objects are the basic entities of reality [6, 7]. Processes are often composed of smaller subunits, but they are not defined by their specific composition. A typical example is a hurricane, which technically consists of a large number of air particles, but is better understood as a collective movement that each individual molecule is only briefly a part of. Process ontology assumes that everything in the world is dynamic first and foremost, and that seemingly stable objects merely appear stable at some limited timescale. Importantly, viewing processes as prior to objects reverses the traditional line of reasoning: one should not think of fundamentally static objects causing dynamic processes, but instead it is fundamentally dynamic processes that stabilize to form (temporarily) static objects. Consequently, the question shifts from asking primarily how change occurs to asking how stable structures can persist over time.

Although process ontology may appear abstract at first glance, it turns out to align remarkably well with cell biology [9, 27]. First, it has become clear that many, if not all, subcellular processes are inherently dynamic. Seemingly stable structures, such as microtubules, septin rings or even bacterial flagellar motors, appear static on a macroscopic scale, but are in fact constantly exchanging their constituents [25, 28, 29]. To assemble such structures, biological systems seem to engage in dynamic, ‘exploratory’ behavior, where different configurations are continuously sampled at a high rate until the desired outcome is found [30, 31, 32]. On top of that, the cytoplasm itself is in continuous flow, displaying currents and eddies that can transport particles and adjust local viscosities [33, 34, 35]. Descriptions focusing on static constituents tend to be less informative for these types of phenomena, since the residence times of individual particles are often much shorter than the lifetime of the collective.

A second important merit of process ontology is the ability to deal with the weakly interacting, heterogenous collectives that are so prominent in cell biology [4]. Consider for instance the aforementioned phenomena of liquid‐liquid phase separation, a process by which proteins and other molecules spontaneously separate into regions of high and low concentration [24, 36]. Although phase separation can be studied from the perspective of stable equilibrium states and the effect of permutations in individual amino acid residues, it is known that phase separated compartments in vivo are often dynamic and heterogeneous [37]. In the cellular context, the occurrence of phase separation does not depend on particular strongly interacting sequences or molecules, but rather on the collective of weak heterogeneous interactions together while being robust to variations in specific interaction strengths [38, 39, 40]. Moreover, phase separation in cells is highly dynamic, as interactions are short‐lived and molecules continuously move within and between the liquid‐like phases [41]. A process perspective naturally suits the description of such phenomena, as it brings the physical process to the core of the explanation rather than individual molecular players.

Although there seems to be general merit to process thinking in cell biology, the consequences of applying a process perspective directly to biological research are not immediately clear. To further concretize the discussion, we now turn our attention to a specific form of cellular organization, being cell polarization in *S. cerevisiae*.

## 3 Identifying Processes in Budding Yeast Polarization

**FIGURE 1:**
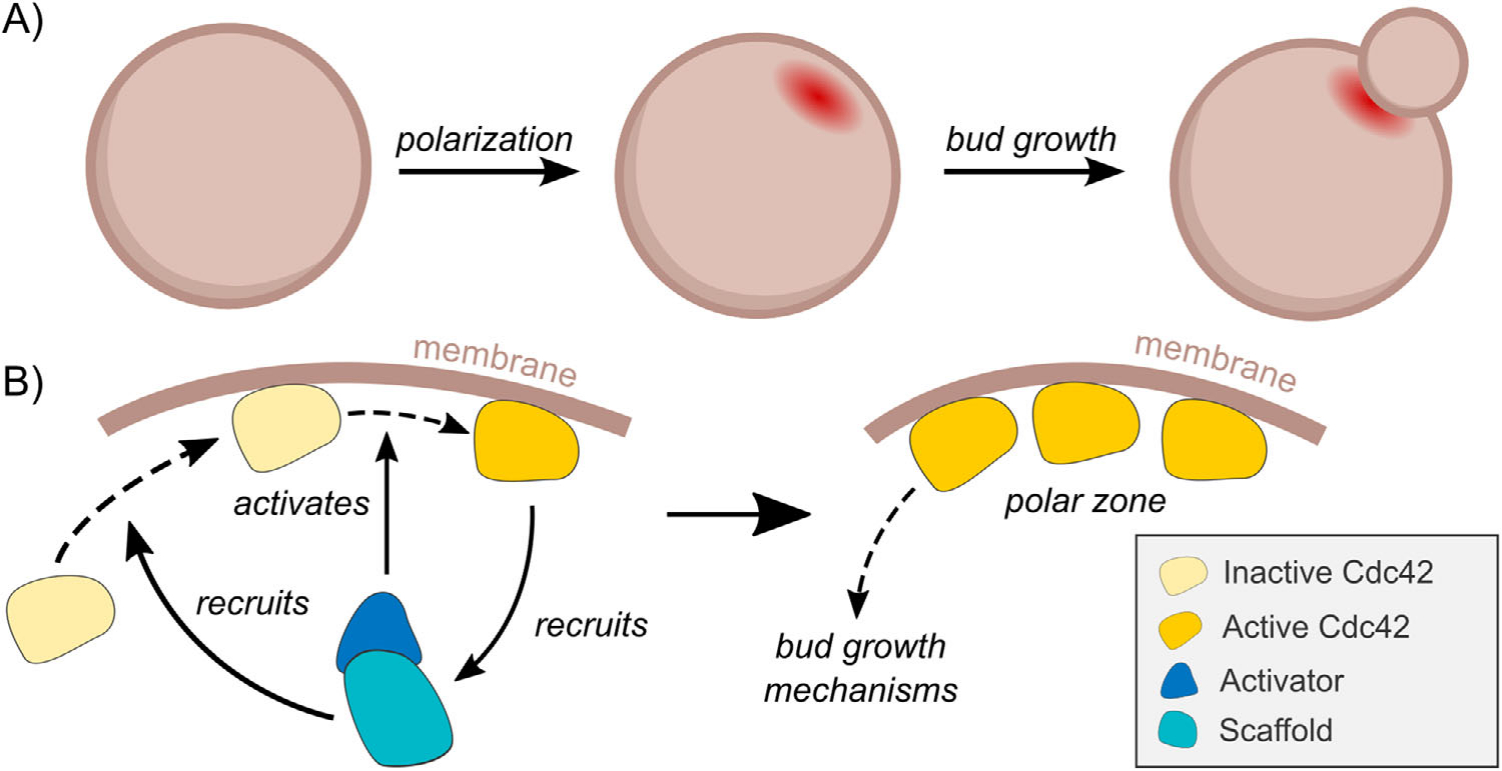
Introduction to polarization in budding yeast. (A) Budding yeast cells need to polarize during their cell division cycle in order to select a unique bud site, from which the daughter cell will grow. (B) Typical (object‐focused) model of polarization by Cdc42, through a positive feedback loop activating Cdc42 at the cell cortex.

Generally speaking, cell polarization refers to the symmetry breaking process through which cells develop distinct internal regions. Establishing polarity is crucial to many different cell types, from developing embryos to epithelial cells to neurons [42, 43, 44]. For the budding yeast *S. cerevisiae*, polarization is required for asymmetric cell division, by specifying a unique membrane site from which the new daughter cell will emerge [45, 46] (Figure 1A). In current molecular descriptions of budding yeast polarity, the problem of bud site selection is often reduced to the accumulation of the signalling GTPase Cdc42, which serves as an attractor for ‘downstream’ components that eventually lead to the assembly of the structural parts required for bud emergence and growth [47]. Cdc42 participates in multiple positive feedback loops that amplify its activation and localization once an initial accumulation has been established, either by internal cellular asymmetry or stochastically [48, 49, 50, 51] (see schematic in Figure 1B). Moreover, Cdc42 is in many contexts an essential protein that has been conserved across different species all the way to mammalian cells [52]. For this reason, current mathematical models of yeast polarity almost exclusively take symmetry breaking by Cdc42 as their main focal point [53, 54, 55, 56, 57]. Evidently, the molecular description of yeast polarity is strongly rooted in an object‐based ontology: molecules and cellular composition are assumed to be static and explanations start from specific proteins and interactions.

**FIGURE 2:**
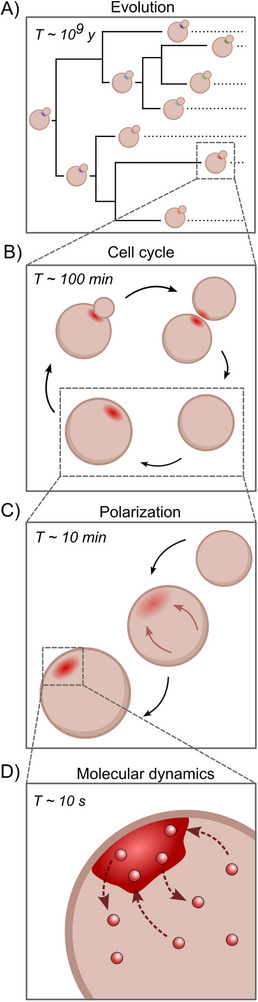
Timescales of processes relevant to budding yeast polarization: evolution taking place over billions of years (A), a cell going through its developmental cycle (B), the symmetry breaking event that establishes polarity (C) and the dynamics of the proteins constituting the polar zone (D).

To provide a processual alternative to this object‐based approach, we will attempt to redescribe yeast polarization in terms of processes as fundamental units. The fundamental processes of polarization can be separated based on relevant timescales, starting with the timescale at which polarity is established inside the cell and subsequently moving to faster and slower processes affecting and underlying polarization. All timescales are visualized in Figure 2.

### 3.1 The Phenomenological Timescale: Symmetry Breaking

The timescale at which polarization has been studied the most is the phenomenological timescale at which it is observed when studying cells under the microscope (Figure 2C). Polarity establishment in budding yeast occurs at a timescale in the order of (tens of) minutes, where exact estimates differ depending on imaging conditions, genetic background and other factors [48, 53, 58, 59]. This timescale defines the process of moving between a cell state in which polarity components are isotropically distributed to a state in which the symmetry is broken and a set of proteins collectively localizes to a concentrated membrane site, thereby initiating bud growth.

As mentioned previously, polarization is often understood through the lens of Cdc42 regulation. However, it has been shown that apart from Cdc42, over one hundred other proteins localize to the site of polarized growth [60]. Although some are well‐characterized interactors of Cdc42, such as those that affect its GTPase cycle, many of these proteins have currently unknown roles in polarization. Besides, although the precise timing of the arrival and depolarization is not known for every component, it is clear that these patterns do not exclusively coincide with the dynamics of Cdc42 [48, 59]. Hence, rather than imagining a concentrated spot of Cdc42 with some of its regulators, the polarity spot might be better represented as a highly heterogeneous and dynamic environment, with a constantly changing constitution. The process at the phenomenological timescale then becomes a flux of effective protein gradients that form and dissolve collectively.

### 3.2 Shorter Timescales: Molecular Dynamics

At shorter timescales, we consider the effect of molecular dynamics on polarization. Importantly, individual molecules themselves are not localizing at the timescale of polarity establishment. The difference in timescales is multiple orders of magnitude: FRAP data has shown that many polarity proteins have residence times at the polar zone in the order of seconds [51, 60, 61]. This means that although the collective has a constant appearance at the phenomenological timescale, the individual constituents are continuously exchanged between the cytoplasm and the polarized spot (Figure 2D).

The dynamicity of the polar zone is further emphasized by the crucial role of GTPases in polarization, not just in budding yeast, but in many cell types and organisms [44, 62, 63, 64]. GTPases are GTP hydrolyzing proteins that often undergo a conformational change when binding to either GTP or GDP, the hydrolyzed version of GTP. Key polarity regulator Cdc42 is a GTPase, which in its GTP‐bound (or ‘active’) state interacts with important bud growth effectors at the membrane [58, 65, 66]. However, continuous cycling between the GTP‐ and GDP‐bound states is essential for polarization: mutants in which Cdc42 is permanently GTP‐bound display severe polarity defects [50, 67]. Cdc42 is far from the only GTPase involved in cell polarity. In fact, different families of GTPases were among the first polarity‐related proteins to be discovered in yeast. Some of them are essential in budding yeast like Cdc42, while others are not. In many cases, the roles of different GTPases are still not fully understood [68].

The importance of GTPase cycles and energy consumption in polarization can be interpreted as another example of exploratory behavior in cellular processes: continuous cycling of components provides opportunity to redirect or correct the direction of polarization, faster than what is possible with highly temporally stable localization. This idea is strengthened by the important role of negative feedback and spot disassembly in polarization, which are crucial to ensure the uniqueness of the polar zone [69, 70]. Moreover, the processual nature of GTPases and their regulation allows for a high level of flexibility in the positioning of the spot, which is for instance important during tracking of chemical signals [71].

Another process occurring at short timescales is the chemical alteration of polarity components by means of post‐translational modifications (PTMs). The effect of PTMs is only beginning to be discovered, but some roles have already been identified in polarization. For instance, important polarity regulator Cdc24 is gradually phosphorylated over the course of polarization, presumably with an inhibitory effect [72, 73]. Moreover, phosphorylation can alter biochemical affinities, which can in turn regulate phase separating behavior of polarized components [74]. In general, many proteins appear to have a multitude of modification sites for different types of PTMs, opening up a range of possible chemical states that can tune protein interactivity and behavior [75].

The observations discussed here suggest that even the polarizing molecules are not strictly static objects, but instead continuously change their properties over time. This argument is strengthened by the fact that even isolated proteins are notoriously difficult to keep stable in a laboratory environment [76]. In that sense, biomolecules might even be considered processes themselves, following the processual view that static structures are more accurately described as temporarily stabilized dynamics [76, 77, 78, 79]. This implies that assessing the role of individual proteins in cellular processes can be deceptive, since the state of a single protein is variable over time and therefore challenging to define statically.

### 3.3 Longer Timescales: Cell Cycle and Evolution

When considering an organism in its entirety, a defining process to consider is its reproductive life cycle [9, 80] (Figure 2B). In the case of asexual reproduction in single celled organisms such as budding yeast, this corresponds to the cell cycle, the repeating pattern by which a cell grows, replicates its DNA and eventually splits into two new entities, which then continue into the next cycle [81]. Throughout this process, a cell exchanges materials with its environment and metabolizes nutrient sources into the energy used to execute cellular functions while simultaneously producing new components to perform these functions.

Polarization is closely tied to the yeast cell cycle in the sense that it needs to occur once and only once during the entire replicative process, with highly constrained timing. For this reason, the initiation of polarization is tightly linked to proteins known as cyclins and their regulators and effectors [82, 83]. Cyclins act as a cellular ‘clock’ and initiate many downstream processes that are time sensitive. In polarization, the presence of cyclins affects the availability of certain proteins in the cytoplasm, for instance by the release of polarity activator Cdc24 from the nucleus through indirect interaction with cyclin‐dependent kinases [84, 85, 86]; presence of Cdc24 is then sufficient to trigger a second stage of polarity amplification [48]. Thus, the available mechanisms of polarization are a function of timing within the cell cycle.

Importantly, since all cells are in constant development, there is no point in the cycle that can be highlighted as the ‘default’ cell state. Moreover, the current state of a cell is in many ways dependent on the history of its ancestors, which can for instance be observed in the differences between polarizing mother and daughter cells and in the effects of replicative aging on division phenotypes [59, 87, 88]. This stresses that any individual polarization event is not happening in isolation but is instead affected by processes happening at larger timescales, a kind of hierarchical relationality that is key to process ontology.

Finally, it is impossible to consider cellular processes without acknowledging their evolutionary history (Figure 2A). Present‐day life forms, including common model organisms like budding yeast have been and are in fact still continuously evolving. This means that the biological systems under study should not be considered static optimized endpoints, but snapshots taken at a somewhat arbitrary point in their evolutionary history. Evolution is continuous, as demonstrated by long‐term laboratory evolution experiments: even in constant environmental conditions, new mutations keep arising in the population [89, 90]. Moreover, evidence is increasingly suggesting that evolution allows for great molecular and phenotypic diversity, even for essential cellular processes that are under strong selection pressure. For instance, centromeric regions are among the fastest evolving genomic regions, despite their interaction with more strongly conserved kinetochore complexes [17]. Additionally, while phenotypes can remain stable under large amounts of genetic variability, the reverse can also be true: genetically homogeneous populations can display significant variances in cellular phenotypes. For instance, mitotic spindle dynamics were found to vary greatly between different natural isolates of *C. elegans*, and even within the same isolate [91]. These observations underline that genes (the objects) are not one‐to‐one correlated with cellular phenotype (the process) and are often less strongly conserved than the processes themselves, which becomes especially apparent at evolutionary timescales.

Returning to fungal polarity, bioinformatic studies have shown great genetic variation between species: sets of polarity proteins can differ significantly even between species that are phylogenetically closely related [15]. Proteins that are essential in budding yeast are non‐essential or even absent in other species, including ‘main regulator’ Cdc42 [92, 93]. At the same time, yeast cells are highly resilient and capable of genome‐wide rearrangements to evolutionarily recover from perturbations, including to polarity [16, 94, 95, 96]. Given this dynamic evolutionary picture, it seems that the current molecular composition of the polarization network in a typical budding yeast cell is better envisioned as just one possible, transient ‘solution’, rather than a fixed, optimal configuration. This may shift the focus from properties of specific constituents to those of the larger process they are a part of.

### 3.4 Consequences of Processes in Yeast Cell Polarity

Restructuring yeast polarity in terms of fundamental processes entails a change in perspective from molecular details to dynamic patterns. As a result, switching to a process‐oriented view comes with some more general consequences. One of the most important consequences of adopting a process ontology is that it moves the *explanandum* from the dynamic to the static. In other words, rather than stillness being the default and movement requiring explanation, a process view assumes dynamism and asks how stable structures can be maintained at certain timescales [6, 9]. This prompts the question how stable polarity actually is in the first place.

The role of polarization in budding yeast is to select a unique membrane site to form a bud. Hence, we are often interested in the final assembly of proteins that determines this site. However, upon closer inspection, it is not clear what this final assembly should look like and if it even exists at all. First of all, while polarity components do localize to form a polarized front, it appears that this front is never final. Rather, gradual depolarization or even oscillations can occur after initial rapid establishment, and peak polarity is not maintained for longer than a minute [69, 73]. Moreover, we have described above how the composition of the polar spot is constantly changing over time, with recruitment of various interactors and structural components that are required for bud emergence. Therefore, instead of a stable spot with static characteristics, the polar zone seems more akin to a dynamic environment shaped to facilitate the structural changes that need to occur for successful cell division.

Much more stable forms of polarization can be found elsewhere in biology, such as polarity in epithelial cells or axis differentiation in embryonic development [44]. In budding yeast polarity, the disassembly of polarized signal at the appropriate time is as essential as its assembly; stable forms of polarity however result in permanent asymmetries that persist for the lifespan of the cell or organism. Here, the question becomes which mechanisms perpetuate the initial asymmetry, which is often established by mechanisms similar to those in budding yeast: in the case of epithelial cells, initial symmetry breaking by several GTPase complexes gives rise to a wealth of different signals that together direct membrane trafficking and further asymmetric deposition of new proteins [97, 98]; and in the case of embryonic development, the primary asymmetry in the first dividing cells eventually results in differentiation into specific body plans [99, 100]. In either case, stability is not a given but needs to be actively propagated or incorporated into subsequent processes to be maintained.

An interesting consequence of the processual perspective in the context of collectivity is that it reverses the questions to be asked, and by that the object of study. By focusing on processes, we are no longer directly concerned with the biological function of genes or proteins separately, but the process itself has a place in a biological system and has evolved under selection in the context of that system. In that sense, the biological requirements or constraints on the process as a whole dictate the constraints on the properties of the individual constituents over time, meaning that components should be understood from the perspective of the collective rather than the other way around. For yeast polarity, this translates to a focus on physical or phenomenological characteristics of the polarized protein composite rather than the roles and interactions of specific proteins.

## 4 Towards Theoretical and Experimental Implementation

Identifying and interpreting the meaning of processes in yeast polarization demonstrates the potential of process ontology as a new lens for understanding biological systems. However, due to the abstraction of the ideas involved, it remains challenging to apply processual views to any biological system in such a way that it can directly inform practical research. Therefore, we will finish with some more concrete implications and examples of incorporating process thought into actual practice, both theoretically and experimentally.

First, it is important to realize that the conceptual implications of a processual perspective, even when somewhat vague, carry significant weight. Science is inevitably driven by underlying worldviews, whether consciously expressed or not. The current dominant worldview might be perceived as an obvious default, which is often the case for reigning paradigms [101, 102], but in reality it is the result of a (mostly subconscious) choice to adopt an object‐based ontology, which then determines how research is practiced and the type of scientific questions that can be asked. Making this choice explicit contributes to a more deliberate approach to scientific problems, and adopting a processual perspective instead opens up a new range of relevant questions and interpretations.

For theoretical work, process ontology invites approaches that focus on dynamicity of the whole. As mentioned previously, thinking in processes implies questioning constancy rather than change. For any cellular system, it can be informative to interrogate the stability of a phenomenon and to assess how this stability is realized, if present at all. As we have seen in yeast polarity, this perspective leads to the realization that the polarized state can never be considered fully stable; consequently, descriptions or theories of polarity should be explicit about which moment in the process they aim to address if the outcome is static, or else account for a dynamic end state. In terms of specific modelling tools, the framework of dynamical systems theory lends itself particularly well for describing dynamic processes, especially because it allows for non‐static attractor states like limit cycles. As an example, studies on cellular signal processing have demonstrated how cells deal with time‐varying or unstable external cues, which prompted the formulation of a novel theoretical framework based on transient dynamics around criticality [103, 104, 105]. Recently much progress has been made in deriving dynamical models top‐down from experimental data rather than constructing them bottom‐up [106, 107, 108]; these approaches are excellent examples of starting from observations at the collective, phenomenological level in order to understand underlying microscopic properties. Finally, some theory development makes explicit mention of process ontology, for instance in work discussing a process approach applied to ecological models and gene regulatory networks [109, 110].

Experimentally, direct application of the process perspective remains more challenging. A significant part of this challenge is caused by the fact that our current molecular toolbox mostly operates at the level of individual genes or proteins, hence, at the object level. While constructing knock‐out cell lines or fluorescently labelling proteins has become exponentially easier over the past decades, methods to interrogate heterogenous collectives, and dynamics are still lagging behind. However, interesting advances have been made in for instance proximity labelling techniques, which allows identification of the protein environment of a cellular structure or collective over time [111]. Moreover, there exists a wide range of live cell fluorescence imaging techniques aimed at probing dynamics, such as typical bleaching recovery measurements like FRAP/FLIP [112] and probes that visualize cellular conditions over time [113, 114, 115], that might be further adapted into process‐oriented approaches. It will be interesting to see if, and if so, how, processual thinking will inspire novel experimental design in the future.

## 5 Conclusion

Process philosophy is becoming more popular within philosophy of the life sciences, but the gap with cell biological research practice remains challenging to bridge. Here, we made an attempt to narrow this gap by applying a processual perspective to a concrete cell biological system, being yeast polarization. This highlights the dynamic interplay of processes at various timescales and stresses the continuous change underlying these processes, while raising questions about perceived stability.

It would be worthwhile to attempt similar application of process philosophy to other cellular phenomena, to further explore the relevance of process thinking in different contexts as well as encountering possible limitations. To this end, one could follow a comparable line of thought as presented here, by first dissecting the phenomena in terms of processes at relevant timescales, abstracted away from object‐like components, and subsequently reformulating questions and observations about the system from the perspective of those fundamental processes. Depending on the system at hand, this may require dominant assumptions to be challenged or reframed. For instance, in the case of metabolism, the sequential picture of metabolite chains catalyzed by specific conserved enzymes is strongly object based, while a description based on flows of metabolites through a changing network would be more processual, and some evidence seems to support the latter perspective [116, 117, 118]. As another example, transcriptional regulation can be treated as a static input‐output relation between a combination of regulatory elements and final transcript abundance, whereas single‐cell studies have demonstrated great underlying variability and dynamicity at the cellular level [119, 120]. A processual description could help detangle the role of such dynamics occurring at different timescales. Apart from conceptual explorations into a variety of systems, the application of process philosophy would greatly benefit from experimental design that is explicitly based on processual thinking, much like current experimental design is predominantly based on an object‐focused view. More development into this area would further improve integration of processual thought within current research practice.

At the moment, integration of process philosophy into the life sciences is still in an early stage, and time will tell if this perspective will yield constructive tools in the long term. Yet, an increasing body of research elicits critical examination of the reductionistic object‐based view of the cell. In that context, processual ontology can provide a compelling alternative.

## Author Contributions

**Marieke Glazenburg**: Conceptualization; visualization; writing – original draft; writing – review & editing. **Liedewij Laan**: Conceptualization; writing – review & editing; supervision.

## Conflicts of Interest

The authors declare no conflicts of interest.

## Data Availability

Data sharing is not applicable to this article as no datasets were generated or analysed during the current study.

## References

[1] J. Huxley , Evolution: The Modern Synthesis Definitive edition (Boston Review 2010): p. 770.

[2] L. H. Hartwell , J. J. Hopfield , S. Leibler , and A. W. Murray , “From Molecular to Modular Cell Biology,” Nature 402, no S6761(1999): C47–C52, 10.1038/35011540. 10591225

[3] S. F. Gilbert and S. Sarkar , “Embracing Complexity: Organicism for the 21st Century,” Developmental Dynamics 219 (2000): 1–9, 10.1002/1097-0177(2000)9999:9999<;::AID-DVDY1036>;3.0.CO;2-A. 10974666

[4] P. A. Vasquez , B. Walker , and K. Bloom , “The Power of Weak, Transient Interactions across Biology: A Paradigm of Emergent Behavior,” Physica D: Nonlinear Phenomena 454 (2023): 133866, 10.1016/j.physd.2023.133866. 38274029 PMC10806540

[5] O. J. Klakegg , “Ontology and Epistemology,” Design Methods and Practices for Research of Project Management (Routledge, 2015).

[6] N. Rescher , Process Metaphysics: An Introduction to Process Philosophy (State University of New York Press, 1996).

[7] J. Seibt , "Ontological Tools for the Process Turn in Biology: Some Basic Notions of General Process Theory," in Everything Flows: Towards a Processual Philosophy of Biology, ed. D. J. Nicholson and J. Dupré (Oxford University Press, 2018), 10.1093/oso/9780198779636.003.0006.

[8] J. Seibt , Process Philosophy, ed. E. N. Zalta and U. Nodelman (Metaphysics Research Lab, Stanford University, 2023), https://plato.stanford.edu/archives/win2023/entries/process‐philosophy/.

[9] J. Dupré and D. J. Nicholson ed., “A Manifesto for a Processual Philosophy of Biology,” in Everything Flows: Towards a Processual Philosophy of Biology (Oxford University Press, 2018), 10.1093/oso/9780198779636.003.0001.

[10] D. J. Weatherall , “Science, Medicine, and the Future: Single Gene Disorders or Complex Traits: Lessons from the Thalassaemias and Other Monogenic Diseases,” Bmj 321, no. 7269 (2000): 1117–1120, 10.1136/bmj.321.7269.1117. 11061733 PMC1118897

[11] M. Wellenreuther and B. Hansson , “Detecting Polygenic Evolution: Problems, Pitfalls, and Promises,” Trends in Genetics 32, no. 3 (2016): 155–164, 10.1016/j.tig.2015.12.004. 26806794

[12] P. Schwille and B. P. Frohn , “Hidden Protein Functions and What They May Teach Us,” Trends in Cell Biology 32, no. 2 (2022): 102–109, 10.1016/j.tcb.2021.09.006. 34654605

[13] D. C. Radisky , M. Stallings‐Mann , Y. Hirai , and M. J. Bissell , “Single Proteins Might Have Dual but Related Functions in Intracellular and Extracellular Microenvironments,” Nature Reviews Molecular Cell Biology 10, no. 3 (2009): 228–234, 10.1038/nrm2633. 19190671 PMC2746016

[14] J. Koehler Leman , P. Szczerbiak , and P. D. Renfrew , “Sequence‐structure‐function Relationships in the Microbial Protein Universe,” Nature Communications 14 (2023): 2351, 10.1038/s41467-023-37896-w. PMC10133388 37100781

[15] E. T. Diepeveen , T. Gehrmann , V. Pourquié , T. Abeel , and L. Laan , “Patterns of Conservation and Diversification in the Fungal Polarization Network,” Genome Biology and Evolution 10, no. 7 (2018): 1765–1782, 10.1093/gbe/evy121. 29931311 PMC6054225

[16] M. M. Glazenburg and L. Laan , “Complexity and Self‐organization in the Evolution of Cell Polarization,” Journal of Cell Science 136, no. 2 (2023): jcs259639, 10.1242/jcs.259639. 36691920

[17] J. Helsen , K. Ramachandran , G. Sherlock , and G. Dey , “Progressive Coevolution of the Yeast Centromere and Kinetochore,” Nature 651 (2026): 1012–1019, 10.1038/s41586-025-09779-1. 41299172 PMC12925627

[18] D. McCusker , “Cellular Self‐organization: Generating Order from the Abyss,” Molecular Biology of the Cell 31, no. 3 (2020): 143–148, 10.1091/mbc.E19-04-0207. 31999511 PMC7001480

[19] R. Wedlich‐Söldner and T. Betz , “Self‐organization: The Fundament of Cell Biology,” Philosophical Transactions of the Royal Society B: Biological Sciences 373, no. 1747 (2018): 20170103, 10.1098/rstb.2017.0103. PMC5904291 29632257

[20] D. J. Nicholson , “The Return of the Organism as a Fundamental Explanatory Concept in Biology,” Philosophy Compass 9, no. 5 (2014): 347–359, 10.1111/phc3.12128.

[21] D. Noble , “A Theory of Biological Relativity: No Privileged Level of Causation,” Interface Focus 2, no. 1 (2012): 55–64, 10.1098/rsfs.2011.0067. 23386960 PMC3262309

[22] X. Fang and J. Wang , “Nonequilibrium Thermodynamics in Cell Biology: Extending Equilibrium Formalism to Cover Living Systems,” Annual Review of Biophysics 49 (2020): 227–246, 10.1146/annurev-biophys-121219-081656. 32375020

[23] X. Fang , K. Kruse , T. Lu , and J. Wang , “Nonequilibrium Physics in Biology,” Reviews of Modern Physics 91, no. 4 (2019): 045004, 10.1103/RevModPhys.91.045004.

[24] A. A. Hyman , C. A. Weber , and F. Jülicher , “Liquid‐Liquid Phase Separation in Biology,” Annual Review of Cell and Developmental Biology 30 (2014): 39–58, 10.1146/annurev-cellbio-100913-013325. 25288112

[25] T. Mitchison and M. Kirschner , “Dynamic Instability of Microtubule Growth,” Nature 312, no. 5991 (1984): 237–242, 10.1038/312237a0. 6504138

[26] J. Halatek , F. Brauns , and E. Frey , “Self‐organization Principles of Intracellular Pattern Formation,” Philosophical Transactions of the Royal Society B: Biological Sciences 373, no. 1747 (2018): 20170107, 10.1098/rstb.2017.0107. PMC5904295 29632261

[27] J. Jaeger and N. Monk , “Everything Flows,” EMBO Reports 16, no. 9 (2015): 1064–1067, 10.15252/embr.201541088. 26276845 PMC4576976

[28] H. Li and V. Sourjik , “Assembly and Stability of Flagellar Motor in Escherichia coli,” Molecular Microbiology 80, no. 4 (2011): 886–899, 10.1111/j.1365-2958.2011.07557.x. 21244534

[29] Y. Oh and E. Bi , “Septin Structure and Function in Yeast and beyond,” Trends in Cell Biology 21, no. 3 (2011): 141–148, 10.1016/j.tcb.2010.11.006. 21177106 PMC3073566

[30] E. Braun , "12—Cell‐state Organization by Exploratory Sloppy Dynamics," in Phenotypic Switching, ed. H. Levine , M. K. Jolly , P. Kulkarni , and V. Nanjundiah (Academic Press, 2020): 305–334.

[31] P. Nalbant and L. Dehmelt , “Exploratory Cell Dynamics: A Sense of Touch for Cells?,” Biological Chemistry 399, no. 8 (2018): 809–819, 10.1515/hsz-2017-0341. 29664730

[32] J. Kondev , M. Kirschner , H. G. Garcia , G. L. Salmon , and R. Phillips , “Biological Processes as Exploratory Dynamics,” Biophysical Journal, (2025), in press, 10.1016/j.bpj.2025.09.009. PMC12924703 40936266

[33] E. Fukuhara , E. Dunkley , A. Bevilacqua , and P. Laurino , “Dynamic Cytoplasm: A Physical Regulator of Enzymatic Function,” Biochemistry 64 (2025): 2699–2711, 10.1021/acs.biochem.5c00212. 40517322 PMC12224306

[34] R. M. Garner , A. T. Molines , J. A. Theriot , and F. Chang , “Vast Heterogeneity in Cytoplasmic Diffusion Rates Revealed by Nanorheology and Doppelgänger Simulations,” Biophysical Journal 122, no. 5 (2023): 767–783, 10.1016/j.bpj.2023.01.040. 36739478 PMC10027447

[35] N. W. Goehring and S. W. Grill , “Cell Polarity: Mechanochemical Patterning,” Trends in Cell Biology 23, no. 2 (2013): 72–80, 10.1016/j.tcb.2012.10.009. 23182746

[36] S. F. Banani , H. O. Lee , A. A. Hyman , and M. K. Rosen , “Biomolecular Condensates: Organizers of Cellular Biochemistry,” Nature Reviews Molecular Cell Biology 18, no. 5 (2017): 285–298, 10.1038/nrm.2017.7. 28225081 PMC7434221

[37] J. K. A. Tom and A. A. Deniz , “Complex Dynamics of Multicomponent Biological Coacervates,” Current Opinion in Colloid Interface Science 56 (2021): 101488, 10.1016/j.cocis.2021.101488.

[38] C. P. Brangwynne , P. Tompa , and R. V. Pappu , “Polymer Physics of Intracellular Phase Transitions,” Nature Physics 11, no. 11 (2015): 899–904, 10.1038/nphys3532.

[39] P. Li , S. Banjade , and H.‐C. Cheng , “Phase Transitions in the Assembly of Multivalent Signalling Proteins,” Nature 483, no. 7389 (2012): 336–340, 10.1038/nature10879. 22398450 PMC3343696

[40] D. Zwicker and L. Laan , “Evolved Interactions Stabilize Many Coexisting Phases in Multicomponent Liquids, ”Proceedings of the National Academy of Sciences 119 (2022), http://arxiv.org/abs/2201.10898. 10.1073/pnas.2201250119 PMC9282444 35867744

[41] S. Alberti , “Phase Separation in Biology,” Current Biology 27, no. 20 (2017): R1097–R1102, 10.1016/j.cub.2017.08.069. 29065286

[42] A. Manninen , “Epithelial Polarity—Generating and Integrating Signals from the ECM with Integrins,” Experimental Cell Research 334, no. 2 (2015): 337–349, 10.1016/j.yexcr.2015.01.003. 25597426

[43] S. Tahirovic and F. Bradke , “Neuronal Polarity,” Cold Spring Harbor Perspectives in Biology 1 (2009): a001644–a001644, 10.1101/cshperspect.a001644. 20066106 PMC2773638

[44] J. P. Campanale , T. Y. Sun , and D. J. Montell , “Development and Dynamics of Cell Polarity at a Glance,” Journal of Cell Science 130, no. 7 (2017): 1201–1207, 10.1242/jcs.188599. 28365593 PMC5399778

[45] B. D. Slaughter , S. E. Smith , and R. Li , “Symmetry Breaking in the Life Cycle of the Budding Yeast,” Cold Spring Harbor Perspectives in Biology 1 (2009): a003384–a003384, 10.1101/cshperspect.a003384. 20066112 PMC2773630

[46] K. E. Miller , P. J. Kang , and H.‐O. Park , “Regulation of Cdc42 for Polarized Growth in Budding Yeast,” Microbial Cell 7, no. 7 (2020): 175–189, 10.15698/mic2020.07.722. 32656257 PMC7328677

[47] E. Bi and H.‐O. Park , “Cell Polarization and Cytokinesis in Budding Yeast,” Genetics 191, no. 2 (2012): 347–387, 10.1534/genetics.111.132886.</bib. 22701052 PMC3374305

[48] K. Witte , D. Strickland , and M. Glotzer , “Cell Cycle Entry Triggers a Switch between Two Modes of Cdc42 Activation during Yeast Polarization,” eLife 6 (2017): e26722, 10.7554/eLife.26722. PMC5536948 28682236

[49] A.‐C. Butty , N. Perrinjaquet , and A. Petit , “A Positive Feedback Loop Stabilizes the Guanine‐nucleotide Exchange Factor Cdc24 at Sites of Polarization,” The EMBO Journal 21, no. 7 (2002): 1565–1576, 10.1093/emboj/21.7.1565. 11927541 PMC125953

[50] J. E. Irazoqui , A. S. Gladfelter , and D. J. Lew , “Scaffold‐mediated Symmetry Breaking by Cdc42p,” Nature Cell Biology 5, no. 12 (2003): 1062, 10.1038/ncb1068. 14625559

[51] T. Freisinger , B. Klünder , and J. Johnson , “Establishment of a Robust Single Axis of Cell Polarity by Coupling Multiple Positive Feedback Loops,” Nature Communications 4 (2013): 1807, 10.1038/ncomms2795. PMC3674238 23651995

[52] D. I. Johnson , “Cdc42: An Essential Rho‐Type GTPase Controlling Eukaryotic Cell Polarity,” Microbiology and Molecular Biology Reviews 63 (1999): 54–105, 10.1128/mmbr.63.1.54-105.1999. 10066831 PMC98957

[53] B. Klünder , T. Freisinger , R. Wedlich‐Söldner , and E. Frey , “GDI‐Mediated Cell Polarization in Yeast Provides Precise Spatial and Temporal Control of Cdc42 Signaling,” PLOS Computational Biology 9, no. 12 (2013): 1003396, 10.1371/journal.pcbi.1003396. PMC3861033 24348237

[54] Y. Liu , J. Xie , H.‐O. Park , and W.‐C. Lo , “Mathematical Modeling of Cell Polarity Establishment of Budding Yeast,” Communications on Applied Mathematics and Computation 6 (2024): 218–235, 10.1007/s42967-022-00240-y.

[55] F. Brauns , L. Iñigo de la Cruz , and W. K.‐G. Daalman , “Redundancy and the Role of Protein Copy Numbers in the Cell Polarization Machinery of Budding Yeast,” Nature Communications 14 (2023): 6504, 10.1038/s41467-023-42100-0. PMC10579396 37845215

[56] A. B. Goryachev and M. Leda , “Many Roads to Symmetry Breaking: Molecular Mechanisms and Theoretical Models of Yeast Cell Polarity,” Molecular Biology of the Cell 28, no. 3 (2017): 370–380, 10.1091/mbc.e16-10-0739. 28137950 PMC5341721

[57] S. Hladyshau , K. Guan , N. Nivedita , B. Errede , D. Tsygankov , and T. C. Elston , “Multiscale Modeling of Bistability in the Yeast Polarity Circuit,” Cells 13, no. 16 (2024): 1358, 10.3390/cells13161358. 39195248 PMC11352540

[58] B. Woods , H. Lai , C.‐F. Wu , T. R. Zyla , N. S. Savage , and D. J. Lew , “Parallel Actin‐Independent Recycling Pathways Polarize Cdc42 in Budding Yeast,” Current Biology 26, no. 16 (2016): 2114–2126, 10.1016/j.cub.2016.06.047. 27476596 PMC5956535

[59] S. Okada , M. Leda , J. Hanna , N. S. Savage , E. Bi , and A. B. Goryachev , “Daughter Cell Identity Emerges from the Interplay of Cdc42, Septins, and Exocytosis,” Developmental Cell 26, no. 2 (2013): 148–161, 10.1016/j.devcel.2013.06.015. 23906065 PMC3730058

[60] J. T. Gao , R. Guimerà , and H. Li , “Modular Coherence of Protein Dynamics in Yeast Cell Polarity System,” Proceedings of the National Academy of Sciences 108, no. 18 (2011): 7647–7652, 10.1073/pnas.1017567108. PMC3088571 21502521

[61] B. D. Slaughter , A. Das , J. W. Schwartz , B. Rubinstein , and R. Li , “Dual Modes of Cdc42 Recycling Fine‐Tune Polarized Morphogenesis,” Developmental Cell 17, no. 6 (2009): 823–835, 10.1016/j.devcel.2009.10.022. 20059952 PMC2805562

[62] S. Etienne‐Manneville and A. Hall , “Rho GTPases in Cell Biology,” Nature 420, no. 6916 (2002): 629, 10.1038/nature01148. 12478284

[63] S.‐T. Sit and E. Manser , “Rho GTPases and Their Role in Organizing the Actin Cytoskeleton,” Journal of Cell Science 124, no. 5 (2011): 679–683, 10.1242/jcs.064964. 21321325

[64] W. M. Bement , A. B. Goryachev , A. L. Miller , and G. von Dassow , “Patterning of the Cell Cortex by Rho GTPases,” Nature Reviews Molecular Cell Biology 25, no. 4 (2024): 290–308, 10.1038/s41580-023-00682-z. 38172611 PMC12706751

[65] J. L. Johnson , J. W. Erickson , and R. A. Cerione , “New Insights into How the Rho Guanine Nucleotide Dissociation Inhibitor Regulates the Interaction of Cdc42 with Membranes,” Journal of Biological Chemistry 284, no. 35 (2009): 23860–23871, 10.1074/jbc.M109.031815. 19581296 PMC2749158

[66] M. Iwase , J. Luo , and S. Nagaraj , “Role of a Cdc42p Effector Pathway in Recruitment of the Yeast Septins to the Presumptive Bud Site,” Molecular Biology of the Cell 17, no. 3 (2006): 1110–1125, 10.1091/mbc.e05-08-0793. 16371506 PMC1382302

[67] A. S. Gladfelter , I. Bose , T. R. Zyla , E. S. G. Bardes , and D. J. Lew , “Septin Ring Assembly Involves Cycles of GTP Loading and Hydrolysis by Cdc42p,” The Journal of Cell Biology 156, no. 2 (2002): 315–326, 10.1083/jcb.200109062. 11807094 PMC2199227

[68] H.‐O. Park and E. Bi , “Central Roles of Small GTPases in the Development of Cell Polarity in Yeast and beyond,” Microbiology and Molecular Biology Reviews 71 (2007): 48–96, 10.1128/MMBR.00028-06. 17347519 PMC1847380

[69] A. S. Howell , M. Jin , C.‐F. Wu , T. R. Zyla , T. C. Elston , and D. J. Lew , “Negative Feedback Enhances Robustness in the Yeast Polarity Establishment Circuit,” Cell 149, no. 2 (2012): 322–333, 10.1016/j.cell.2012.03.012. 22500799 PMC3680131

[70] C.‐F. Wu and D. J. Lew , “Beyond Symmetry‐breaking: Competition and Negative Feedback in GTPase Regulation,” Trends in Cell Biology 23, no. 10 (2013): 476–483, 10.1016/j.tcb.2013.05.003. 23731999 PMC3783641

[71] J. B. Kelley , G. Dixit , J. B. Sheetz , S. P. Venkatapurapu , T. C. Elston , and H. G. Dohlman , “RGS Proteins and Septins Cooperate to Promote Chemotropism by Regulating Polar Cap Mobility,” Current Biology 25, no. 3 (2015): 275–285, 10.1016/j.cub.2014.11.047. 25601550 PMC4318785

[72] K. D. Moran , H. Kang , and A. V. Araujo , “Cell‐cycle Control of Cell Polarity in Yeast,” Journal of Cell Biology 218, no. 1 (2018): 171–189, 10.1083/jcb.201806196. 30459262 PMC6314536

[73] C.‐C. Kuo , N. S. Savage , H. Chen , C.‐F. Wu , T. R. Zyla , and D. J. Lew , “Inhibitory GEF Phosphorylation Provides Negative Feedback in the Yeast Polarity Circuit,” Current Biology 24, no. 7 (2014): 753–759, 10.1016/j.cub.2014.02.024. 24631237 PMC4018745

[74] Y. Xie and Y. Miao , “Polarisome Assembly Mediates Actin Remodeling during Polarized Yeast and Fungal Growth,” Journal of Cell Science 134, no. 1 (2021): jcs247916, 10.1242/jcs.247916. 33419950

[75] N. Šoštarić and V. Noort , “Molecular Dynamics Shows Complex Interplay and Long‐range Effects of Post‐translational Modifications in Yeast Protein Interactions,” PLOS Computational Biology 17, no. 5 (2021): 1008988, 10.1371/journal.pcbi.1008988. PMC8143416 33979327

[76] S. Güttinger , “Process and Practice: Understanding the Nature of Molecules,” HYLE: International Journal for Philosophy of Chemistry 27 (2021): 47‐66.

[77] S. Guttinger , "A Process Ontology for Macromolecular Biology," in Everything Flows: Towards a Processual, ed. D. J. Nicholson and J. Dupré (Oxford University Press, 2018), 10.1093/oso/9780198779636.003.0015.

[78] F. Alassia , “A Process Ontology Approach in Biochemistry: The Case of GPCRs and Biosignaling,” Foundations of Chemistry 24, no. 3 (2022): 405–422, 10.1007/s10698-022-09443-w.

[79] R. L. Stein , “Towards a Process Philosophy of Chemistry,” HYLE: International Journal for Philosophy of Chemistry 10, no. 1 (2004): 5–22.

[80] J. Jaeger , "The Fourth Perspective: Evolution and Organismal Agency," in Organization in Biology, ed. M. Mossio (Springer International Publishing, 2024): 159–186, 10.1007/978-3-031-38968-9_8.

[81] A. A. Duina , M. E. Miller , and J. B. Keeney , “Budding Yeast for Budding Geneticists: A Primer on the Saccharomyces Cerevisiae Model System,” Genetics 197 (2014): 33–48, 10.1534/genetics.114.163188. 24807111 PMC4012490

[82] D. J. Lew and S. I. Reed , “Cell Cycle Control of Morphogenesis in Budding Yeast,” Current Opinion in Genetics Development 5, no. 1 (1995): 17–23, 10.1016/S0959-437X(95)90048-9. 7749320

[83] D. Pirincci Ercan , F. Chrétien , P. Chakravarty , H. R. Flynn , A. P. Snijders , and F. Uhlmann , “Budding Yeast Relies on G1 Cyclin Specificity to Couple Cell Cycle Progression with Morphogenetic Development,” Science Advances 7, no. 23 (2021): abg0007, 10.1126/sciadv.abg0007. PMC8177710 34088668

[84] M. Peter , A. Gartner , J. Horecka , G. Ammerer , and I. Herskowitz , “FAR1 links the Signal Transduction Pathway to the Cell Cycle Machinery in Yeast,” Cell 73, no. 4 (1993): 747–760, 10.1016/0092-8674(93)90254-N. 8500168

[85] Y. Shimada , M.‐P. Gulli , and M. Peter , “Nuclear Sequestration of the Exchange Factor Cdc24 by Far1 Regulates Cell Polarity during Yeast Mating,” Nature Cell Biology 2, no. 2 (2000): 117–124, 10.1038/35000073. 10655592

[86] S. Henchoz , Y. Chi , B. Catarin , I. Herskowitz , R. J. Deshaies , and M. Peter , “Phosphorylation‐ and Ubiquitin‐dependent Degradation of the Cyclin‐dependent Kinase Inhibitor Far1p in Budding Yeast,” Genes Development 11, no. 22 (1997): 3046–3060, 10.1101/gad.11.22.3046. 9367986 PMC316705

[87] P. J. Kang , K. E. Miller , J. Guegueniat , L. Beven , and H.‐O. Park , “The Shared Role of the Rsr1 GTPase and Gic1/Gic2 in Cdc42 Polarization,” Molecular Biology of the Cell 29, no. 20 (2018): 2359–2369, 10.1091/mbc.E18-02-0145. 30091649 PMC6233053

[88] E. J. Yang , W. M. Pernice , and L. A. Pon , “A Role for Cell Polarity in Lifespan and Mitochondrial Quality Control in the Budding Yeast Saccharomyces Cerevisiae,” Iscience 3 (2022): 25, 10.1016/j.isci.2022.103957. PMC8914336 35281729

[89] R. E. Lenski , “Experimental Evolution and the Dynamics of Adaptation and Genome Evolution in Microbial Populations,” The ISME Journal 11, no. 10 (2017): 2181, 10.1038/ismej.2017.69. 28509909 PMC5607360

[90] M. S. Johnson , S. Gopalakrishnan , and J. Goyal , “Phenotypic and Molecular Evolution across 10,000 Generations in Laboratory Budding Yeast Populations,” Elife 10 (2021): 63910, 10.7554/eLife.63910. PMC7815316 33464204

[91] R. Farhadifar , C. F. Baer , and A.‐C. Valfort , “Scaling, Selection, and Evolutionary Dynamics of the Mitotic Spindle,” Current Biology 25 (2015): 732–740, 10.1016/j.cub.2014.12.060. 25683802 PMC10504684

[92] E. T. Diepeveen , L. I. de la Cruz , and L. Laan , “Evolutionary Dynamics in the Fungal Polarization Network, a Mechanistic Perspective,” Biophysical Reviews 9, no. 4 (2017): 375–387, 10.1007/s12551-017-0286-2. 28812259 PMC5578929

[93] M. Mahlert , L. Leveleki , A. Hlubek , B. Sandrock , and M. Bölker , “Rac1 and Cdc42 Regulate Hyphal Growth and Cytokinesis in the Dimorphic Fungus Ustilago maydis,” Molecular Microbiology 59, no. 2 (2006): 567–578, 10.1111/j.1365-2958.2005.04952.x. 16390450

[94] E. Kingma and L. Laan , “Global genetic rewiring during compensatory evolution in the yeast polarity network,” EMBP Reports 27 (2026): 1414–1436, 10.1038/s44319-026-00709-4. PMC13022240 41699154

[95] G. Liu , M. Y. J. Yong , M. Yurieva et al., “Gene Essentiality is a Quantitative Property Linked to Cellular Evolvability,” Cell 163, no. 6 (2015): 1388–1399, 10.1016/j.cell.2015.10.069. 26627736

[96] L. Laan , J. H. Koschwanez , and A. W. Murray , “Evolutionary Adaptation after Crippling Cell Polarization Follows Reproducible Trajectories,” Elife 4 (2015): e09638, 10.7554/eLife.09638. PMC4630673 26426479

[97] G. Apodaca , L. I. Gallo , and D. M. Bryant , “Role of Membrane Traffic in the Generation of Epithelial Cell Asymmetry,” Nature Cell Biology 14, no. 12 (2012): 1235–1243, 10.1038/ncb2635. 23196841 PMC3771702

[98] S. P. Ngok , W.‐H. Lin , and P. Z. Anastasiadis , “Establishment of Epithelial Polarity—GEF Who's Minding the GAP?,” Journal of Cell Science 127, no. 15 (2014): 3205–3215, 10.1242/jcs.153197. 24994932 PMC4117226

[99] M. Levin , “Left–right Asymmetry in Embryonic Development: A Comprehensive Review,” Mechanisms of Development 122, no. 1 (2005): 3–25, 10.1016/j.mod.2004.08.006. 15582774

[100] Á. Raya and J. C. I. Belmonte , “Left–right Asymmetry in the Vertebrate Embryo: From Early Information to Higher‐level Integration,” Nature Reviews Genetics 7, no. 4 (2006): 283–293, 10.1038/nrg1830. 16543932

[101] T. S. Kuhn , The Structure of Scientific Revolutions., (University of Chicago Press, 1962).

[102] C. A. Layman and A. L. Rypel , “Beyond Kuhnian Paradigms: Normal Science and Theory Dependence in Ecology,” Ecology and Evolution 13, no. 7 (2023): 10255, 10.1002/ece3.10255. PMC10318613 37408635

[103] D. Koch , A. Nandan , G. Ramesan , and A. Koseska , “Biological Computations: Limitations of Attractor‐based Formalisms and the Need for Transients,” Biochemical and Biophysical Research Communications 720 (2024): 150069, 10.1016/j.bbrc.2024.150069. 38754165

[104] A. Nandan , A. Das , R. Lott , and A. Koseska , “Cells Use Molecular Working Memory to Navigate in Changing Chemoattractant Fields,” Elife 11 (2022): 76825, 10.7554/eLife.76825. PMC9282860 35666122

[105] A. Nandan and A. Koseska , “Non‐asymptotic Transients away from Steady States Determine Cellular Responsiveness to Dynamic Spatial‐temporal Signals,” PLOS Computational Biology 19, no. 8 (2023): 1011388, 10.1371/journal.pcbi.1011388. PMC10449117 37578988

[106] I. Seim and S. W. Grill , “Empirical Methods That Provide Physical Descriptions of Dynamic Cellular Processes,” Biophysical Journal 124 (2025): 861–875, 10.1016/j.bpj.2024.12.003. 39639772 PMC11947468

[107] T. Brandstätter , “Data‐Driven Theory Reveals Protrusion and Polarity Interactions Governing Collision Behavior of Distinct Motile Cells,” PRX Life 3, no. 3 (2025), 10.1103/3hhj-rt1n.

[108] D. B. Brückner and C. P. Broedersz , “Learning Dynamical Models of Single and Collective Cell Migration: A Review,” Reports on Progress in Physics 87, no. 5 (2024): 056601, 10.1088/1361-6633/ad36d2. 38518358

[109] C. Gaucherel , “Why and How to Use Process Philosophy in Everyday Ecology and Biology?,” Acta Biotheoretica 73, no. 4 (2025): 14, 10.1007/s10441-025-09504-5. 40928590

[110] J. DiFrisco and J. Jaeger , “Beyond Networks: Mechanism and Process in Evo‐devo,” Biology Philosophy 34, no. 6 (2019): 54, 10.1007/s10539-019-9716-9.

[111] W. Qin , K. F. Cho , P. E. Cavanagh , and A. Y. Ting , “Deciphering Molecular Interactions by Proximity Labeling,” Nature Methods 18, no. 2 (2021): 133–143, 10.1038/s41592-020-01010-5. 33432242 PMC10548357

[112] H. C. Ishikawa‐Ankerhold , R. Ankerhold , and G. P. C. Drummen , “Advanced Fluorescence Microscopy Techniques—FRAP, FLIP, FLAP, FRET and FLIM,” Molecules 17 (2012): 4047–4132, 10.3390/molecules17044047. 22469598 PMC6268795

[113] J. Han and K. Burgess , “Fluorescent Indicators for Intracellular pH,” Chemical Reviews 110, no. 5 (2010): 2709–2728, 10.1021/cr900249z. 19831417

[114] H. Wang , Z. Feng , S. J. Del Signore , A. A. Rodal , and B. Xu , “Active Probes for Imaging Membrane Dynamics of Live Cells with High Spatial and Temporal Resolution over Extended Timescales and Areas,” Journal of the American Chemical Society 140, no. 10 (2018): 3505–3509, 10.1021/jacs.7b13307. 29481071 PMC5858877

[115] S. Lecinski , J. W. Shepherd , and K. Bunting , “Correlating Viscosity and Molecular Crowding with Fluorescent Nanobeads and Molecular Probes: In Vitro and in Vivo,” Interface Focus 12, no. 6 (2022): 20220042, 10.1098/rsfs.2022.0042. 36330320 PMC9560789

[116] M. A. Huynen , T. Dandekar , P. Bork , M. A. Huynen , T. Dandekar , and P. Bork , “Variation and Evolution of the Citric‐acid Cycle: A Genomic Perspective,” Trends in Microbiology 7, no. 7 (1999): 281–291, 10.1016/S0966-842X(99)01539-5. 10390638

[117] C. B. Hubert and L. P. S. de Carvalho , “Convergence and Divergence in the Metabolic Network of *Mycobacterium Tuberculosis* ,” Current Opinion in Systems Biology 28 (2021): 100384, 10.1016/j.coisb.2021.100384.

[118] R. C. Law , A. Lakhani , S. O'Keeffe , S. Erşan , and J. O. Park , “Integrative Metabolic Flux Analysis Reveals an Indispensable Dimension of Phenotypes,” Current Opinion in Biotechnology 75 (2022): 102701, 10.1016/j.copbio.2022.102701. 35278746 PMC9177643

[119] T. C. Voss and G. L. Hager , “Dynamic Regulation of Transcriptional States by Chromatin and Transcription Factors,” Nature Reviews Genetics 15, no. 2 (2014): 69–81, 10.1038/nrg3623. PMC6322398 24342920

[120] M. Furlan , S. de Pretis , and M. Pelizzola , “Dynamics of Transcriptional and Post‐transcriptional Regulation,” Briefings in Bioinformatics 22, no. 4 (2021): bbaa389, 10.1093/bib/bbaa389. 33348360 PMC8294512

